# Prevalence and Molecular Characterization of Shiga Toxin-Producing *Escherichia coli* from Food and Clinical Samples

**DOI:** 10.3390/pathogens12111302

**Published:** 2023-10-31

**Authors:** Khulud Alotaibi, Ashraf A. Khan

**Affiliations:** 1 Division of Microbiology, National Center for Toxicological Research, United States Food and Drug Administration, Jefferson, AR 72079, USA; kholoudsafar@gmail.com; 2 Department of Biology, University of Arkansas at Little Rock, Little Rock, AR 72205, USA

**Keywords:** *Escherichia coli*, STEC, foodborne pathogens, clinical STEC, *stx*

## Abstract

Shiga toxin-producing *Escherichia coli* (STEC) is one of the most prominent food-borne pathogens in humans. The current study aims to detect and to analyze the virulence factors, antibiotic resistance, and plasmid profiles for forty-six STEC strains, isolated from clinical and food strains. Pulsed-field gel electrophoresis (PFGE) was used to determine the genetic relatedness between different serotypes and sources of samples. The clinical samples were found to be resistant to Nb (100%), Tet (100%), Amp (20%), SXT (15%), and Kan (15%) antibiotics. In contrast, the food strains were found to be resistant to Nb (100%), Tet (33%), Amp (16.6%), and SXT (16.6%) antibiotics. The PFGE typing of the forty-six isolates was grouped into more than ten clusters, each with a similarity between 30% and 70%. Most of the isolates were found positive for more than five virulence genes (*eae*, *hlyA*, *stx1*, *stx2*, *stx2f*, *stx2c*, *stx2e*, *stx2*, *nelB*, *pagC*, *sen*, *toxB*, *irp*, *efa*, and *efa1*). All the isolates carried different sizes of the plasmids. The isolates were analyzed for plasmid replicon type by PCR, and 72.5% of the clinical isolates were found to contain X replicon-type plasmid, 50% of the clinical isolates contained FIB replicon-type plasmid, and 17.5% of the clinical isolates contained Y replicon-type plasmid. Three clinical isolates contained both I1 and Hi1 replicon-type plasmid. Only two food isolates contained B/O and W replicon-type plasmid. These results indicate that STEC strains have diverse clonal populations among food and clinical strains that are resistant to several antimicrobials. In conclusion, our findings indicate that food isolates of STEC strains harbor virulence, antimicrobial resistance, plasmid replicon typing determinants like those of other STEC strains from clinical strains. These results suggest that these strains are unique and may contribute to the virulence of the isolates. Therefore, surveillance and characterization of STEC strains can provide useful information about the prevalence of STEC in food and clinical sources. Furthermore, it will help to identify STEC serotypes that are highly pathogenic to humans and may emerge as a threat to public health.

## 1. Introduction

STEC strains are the most prevalent foodborne pathogens, which can cause serious illness in humans [1]. Among them, STEC O157:H7 is a prominent enteropathogen that can cause outbreaks of haemolytic uremic syndrome (HUS) and hemorrhagic colitis (HC); STEC O157:H7 is classified as the most important serotype in relation to human infection, whereas non-O157 cases are increasingly reported [2,3]. The most well-recognized top five STEC pathogens are O157, O26, O103, O111, and O145 [4]. STEC is contracted and transmitted host to host via contaminated food, as well as from human and water surfaces by the fecal–oral route. [5]. The intestinal tracts of animals, especially cattle, can act as reservoirs and transmit STEC [6]. All STEC strains produce one of the most viral pathogenicity factors encoded by a lambdoid bacteriophage, the Shiga toxin (Stx) [7]. Stx can be classified into two types, Stx1 and Stx2, which are encoded by *stx1* and *stx2*, respectively. The most common virulence gene *stx2* can be classified into seven subtypes: *stx2a*, *stx2b*, *stx2c*, *stx2d, stx2e*, *stx2f*, and *stx2g* [6,8]. The *stx1*, *stx2,* and subtypes *stx2a*, *stx2c*, and *stx2d* of STEC strains are more often associated with infections of HC and HUS [9,10]. The *eae* gene, found on the locus of enterocyte effacement (LEE), encodes intimin, which is an adhesin involved in gut colonization. LEE-positive STEC is expected to provoke HUS or HC more frequently than LEE-negative STEC. These virulence genes have been detected in outbreaks over the last several years [11,12,13]. The plasmid profile analysis method has been helpful for the characterization of many different types of STEC. The plasmid profiles of different serotypes of Shiga toxin indicate that large plasmids may carry virulence genes, which increases the bacteria’s virulence potential. There are several STEC strains that usually carry different plasmid types that play important roles in intestinal diseases [14,15]. 

The antibiotic resistance of *E. coli* in the USA has increased over the years [16]. Inadvertent misuse of antibiotics to treat humans and to promote food–animal growth may have allowed antimicrobial resistance in bacteria [17,18]. Recent studies have noted that antimicrobials, including tetracycline, cephalosporins, and penicillin, have significantly contributed to the emergence and dissemination of antimicrobial-resistant STEC. Prudent use of antibiotics to treat STEC infections is necessary, since STEC can produce Stx, causing HUS in humans [19].

In the present study, a detailed analysis of the molecular characteristics of STEC strains isolated from clinical and food samples was performed. The aims were to characterize these strains by antimicrobial resistance pattern and to determine the presence of factors associated with STEC virulence genes by studying the plasmid replicon types. The strains were compared at the molecular level using molecular techniques such as PFGE, as well as virulence gene and plasmid replicon typing profiles to evaluate and to help understand the molecular epidemiology of those *E. coli* strains that are important for surveillance of STEC isolated from clinical and food strains.

## 2. Materials and Methods

### 2.1. Bacterial Strains

All (*n* = 6) strains of food were obtained from the FDA Arkansas Regional Laboratory and the (*n* = 40) clinical strains were obtained from the Arkansas Department of Health, during the last ten years.

### 2.2. DNA Isolation from Bacteria

All bacterial strains were grown as described in previous studies [14,15]. A single colony of each STEC strain was grown overnight in 5 mL Luria Bertani broth at 37 °C with shaking. Total bacterial DNA was extracted using blood and tissue kits according to the manufacturer’s specifications (DNeasy Blood & Tissue Kit, Qiagen, Germantown, TN, USA) and stored at −20 °C.

### 2.3. Serotyping of STEC Strains

STEC strains were serotyped at the Department of Food Science, *E. coli* reference Center, Penn State, College of Agriculture Science, University Park, PA, USA.

### 2.4. Antimicrobial Susceptibility Testing by Disk Diffusion

Antimicrobial susceptibility was tested for seven antimicrobials on 46 STEC isolates using the disk diffusion method on Mueller–Hinton agar plates (Merck, Darmstadt, Germany). Antimicrobial agents tested included ampicillin (10 µg), gentamicin (10 µg), nalidixic acid (30 µg), trimethoprim-sulfamethoxazole (1.25–23.75 µg), tetracycline (30 µg), kanamycin (30 µg), and novobiocin (5 µg). The results were interpreted according to the Clinical and Laboratory Standards Institute [20].

### 2.5. Pulsed-Field Gel Electrophoresis (PFGE)

Bacterial cultures were grown overnight on Luria Bertani agar plates at 37 °C. PFGE was performed following a procedure described by the Centers for Disease Control (CDC) (https://www.cdc.gov/pulsenet/pdf/ecoli-shigella-salmonella-pfge-protocol-508c.pdf, accessed on 2 February 2023). Briefly, the plugs were digested with the restriction enzyme *XbaI* (New England Biolabs, Ipswich, MA, USA). *Salmonella* Braenderup strain H9812 PulseNet standard was used as a molecular marker after digestion with *XbaI* enzyme. Electrophoresis was performed at 6 V/cm with 2.2–54.2 s linear ramp time for 19 h. Fingerprinting profiles were examined using the BioNumerics software with the Dice coefficient and clustering was based on the unweighted pair group average method (UPGA).

### 2.6. Identification of Shiga-Toxin Subtype and Other Genes Associated with Virulence

All strains of STEC were screened for fifteen virulence genes by a polymerase chain reaction (PCR) method. The primers used for this study are listed in Table 1. PCR kits (Tag DNA Polymerase, Qiagen, Germantown, MD, USA) were used with 1 μL of template DNA. The PCR amplification conditions were different for each primer [21,22,23,24]. The PCR products were analyzed by electrophoresis on 1.2% agarose gels in 1 × TAE with Red Safe nucleic acid gel staining at 66 V for 90 min.

### 2.7. Plasmid Profiling

Plasmid DNA was prepared from 3 mL overnight cultures of bacteria grown in Luria Bertani broth (Difco Laboratories, Detroit, MI, USA) by using the alkaline lysis protocol [25]. Plasmid DNA patterns were obtained by electrophoresis on 1% agarose gels in TAE buffer for seven hours, then stained with ethidium bromide, and observed under UV light.

### 2.8. PCR Detection of Plasmid Replicon Typing

Eighteen pairs of primers were used to screen the plasmid incompatibility (Inc) types by using a simplex PCR method [26]. All the primers are listed in Table 2. The conditions of the PCR amplification were 5 min at 94 °C, 30 cycles of 30 s at 94 °C, 30 s at 60 °C, 90 s at 72 °C, and a final extension of 5 min at 72 °C. Amplified PCR products were run by agarose gel electrophoresis on 1.2% agarose gels in 1 × TAE buffer at 66 V for 90 min.

## 3. Results

**Figure 1:**
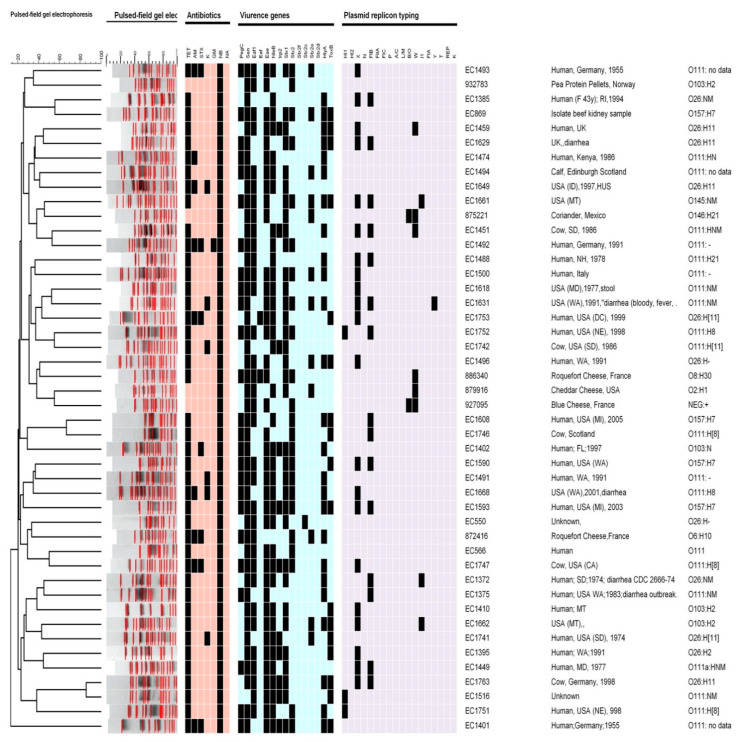
Dendrogram generated by using the BioNumerics software, showing the clusters of pulsed-field gel electrophoresis (PFGE) analysis of the Shiga toxin-producing *Escherichia coli* (STEC) isolates (*n* = 46), the serotypes, virulence genes, plasmid replicon typing profiles, and antibiotics profiles. All data were based on the presence (black squares) and absence (other colors) of the genes investigated.

Serotypes: The most prevalent serotype was O111:HNM (21 out of 46 clinical isolates). In clinical strains (*n* = 46), four isolates were *E. coli* serotype O157:H7, 11 isolates were serotype O26:H11, three isolates were serotype O145:NM, and one isolate was O103:NM. For food strains, the serotypes were O2:H1, O146:H21, NEG: +, O6:H10, O8:H30, and O103:H2. Data and the source for each strain are described in Figure 1.

**Antibiotic profile:** The antimicrobial susceptibility by disk diffusion profile showed that all (*n* = 46) of the strains were resistant to one or more of the antimicrobials tested (Figure 1). The most resistant phenotypes were attributed to novobiocin (100%); 20% of clinical isolates and one food isolate were resistant to ampicillin; 15% of clinical isolates were resistant to kanamycin; all the food strains were sensitive. One of the food isolates and 15% of the clinical strains were resistant to trimethoprim-sulfamethoxazole. None of the strains were resistant to nalidixic acid and only one strain from the clinical strains was resistant to gentamicin. All isolates collected from clinical and 33% from food were resistant to tetracycline.

**PFGE**: Among all strains of STEC O157 and other serotypes from clinical and food strains, all the isolates were singletons and unique PFGE types. Figure 1 shows more than 20 clusters of PFGE. All the clinical and food strains had 18 bands, with one exception, the strain from food that had 22 bands. The most similarity among the isolates was 85% between two strains from clinical isolates, while the least similarity was 15%. The cluster similarity between clinical and food was 35%. Overall, the data demonstrated very low similarity among these strains. One O157:H7 strain had up to 60% similarity with O26:NM; while another strain, i.e., O157:H7, had up to 80% similarity with 111:H8. Most similarities were between two serotypes, O26:H11 and O111:NM, which were 85% of the strains from clinical isolates, and O2:H1 and NEG:+, which were 40% of strains from food isolates. Food and clinical strains’ highest similarity was 40% between O146:H21 and O145:NM serotypes. 

**Virulence genes:** All food isolates (100%) and 48% of clinical isolates showed the presence of the *stx1* virulence gene; 75% of clinical isolates and 66% of food isolates were positive for *stx2*. Only one strain from clinical strains showed the presence of the *stx2c* gene; ~22% of strains from clinical isolates and 50% of food strains were positive for *stx2e*. The virulence genes *stx2f* and *stx2d* were not found in any strains.

The *hlyA* gene was present in 62.5% of the clinical isolates and in all of the food isolates. All the food strains and 85% of the clinical strains showed the presence of the *eae* gene, and 42.5% of the clinical strains and all food strains showed the presence of *toxB.* The *nleB* gene was present in 87.5% of the clinical strains and one (1.6%) food strain. While all the food strains were negative for *Irp2*, 35% of the clinical strains showed the presence of this gene. These results indicate that all the food strains and 97.5% of the clinical strains contain the *sen* gene. Among the food strains, 37.5% were positive for *pegC*. All the food strains and 67.5% of the clinical strains were positive for *efa1*. One strain from clinical strains also had the *efa* gene. Together, these results suggest that the distribution of virulence genes are different in clinical and food isolates. 

**Plasmid:** In all the strains, plasmid profiling experiments identified several mega, large, and small plasmids ranging in size from 80 kb to 20 kb. Each strain carried two or more large mega and small plasmids. Future nucleotide sequencing of plasmids will be useful in determining the incompatibility groups of the plasmids. The data suggest that plasmid profile analysis and typing can be useful in discriminating between isolates from different sources. 

**Plasmid replicon typing:** Eighteen different primers of plasmid replicons were screened by PCR in this study, as listed in Table 2. HI2, N, FIIA, FIC, P, A/C, L/M, FIA, T, REP, and K plasmid replicons/genes were not found in any of the strains (Figure 1). Figure 1 shows that 73% of the clinical strains contained the X gene while none of the food strains showed the presence of this gene. None of the food isolates contained the HI1 gene; however, the HI1 gene was found in 7.5% of the clinical isolates. In addition, 83% of the food strains and 5% of the clinical strains showed the presence of the W plasmid typing gene. The B/O plasmid typing gene was not present in any clinical strains but was present in 33% of food isolates. FIB, Y and I1 genes were found in 33, 2.5, and 7.5% of the clinical strains, respectively. None of the food strains contained the Y and I1 genes.

## 4. Discussion

STEC causes hemorrhagic colitis, diarrhea, and bloody diarrhea in those infected. STEC infections are also a leading cause of numerous foodborne illnesses [27] and frequently result in haemolytic uremic syndrome (HUS), which is a life-threatening condition characterized by hemolytic anemia, thrombocytopenia, and renal failure [28]. STEC modes of infection result in sporadic cases of disease or outbreaks that can involve up to several thousand infected individuals [29]. This study investigated the molecular characterization of STEC strains by determining the virulence genes, antibiotic resistance profiles, and other genes associated with HUS. These molecular factors play important roles in determining the pathogenicity of STEC, thus impacting public health. 

This study analyzed the plasmid profiles in clinical and food strains collected from the FDA Arkansas Regional Laboratory and Arkansas Department of Health, during the last ten years. Our study demonstrated the presence of mega, large, and small plasmids in both groups of strains. These data support previous findings [15] that have indicated that all strains from clinical and food strains carry mega, large, and small plasmids. 

The antimicrobial resistance profile of STEC displayed a valuable indicator for assessing the status of emerging multidrug-resistant STEC strains. In this study, some strains possessed resistance to more than one antibiotic STEC isolate collected from the clinical and food strains. In agreement with previous reports, all isolates in this study were sensitive to nalidixic acid and resistant to novobiocin [30]. Each year, different species of *E. coli* become resistant to several antibiotics [31,32]. In this investigation, multiple instances of antimicrobial resistance to novobiocin and tetracycline were observed. Tetracycline was initially used as an antimicrobial in food animals, with overuse resulting in high rates of resistance [33]. In this study, clinical and food strains displayed resistance to tetracycline ranging from 100 to 33.3%, in agreement with previous studies [34,35].

STEC strains in this study also showed resistance to other antimicrobials such as trimethoprim-sulfamethoxazole, kanamycin, ampicillin, and gentamicin. While all strains tested were sensitive to nalidixic acid and gentamicin, one clinical strain was also resistant to gentamicin. Among the clinical strains, 20% showed resistance to ampicillin,15% to kanamycin and trimethoprim-sulfamethoxazole, and one food strain was resistant to ampicillin. Other food strains were relatively sensitive to all antibiotics used, which was similar to previous findings [36,37]. 

The PFGE technique remains to be a good molecular tool for finding differences and similarities of pathogenic bacteria [38]. Based on the combined genotype and PFGE profiles, human and food O157:H7 can be distinguished from other serotype isolates used in this study. The isolates were grouped into several main clusters, with some clusters including human and food isolates that were loosely similar. These data are similar to a previous study [39].

Pathogenicity profiles play significant roles in Shiga toxins and are regulated by the production of *stx* genes (*stx1* and *stx2*). These Stx serotypes and other virulence factors are generally associated with infections, since diarrhea or HUS, for sure, are not asymptomatic [1]. In this study, genetic characterization of the isolates with regard to the presence of *stx1* and *stx2* genes was performed by PCR. *Stx1* and *stx2* genes were found in from 75% to 50% of both groups. Based on the PCR profiles, these strains should be considered potentially pathogenic for humans. Each food strain has either one or both *stx1* and *stx2* genes, which are often the major contributors resulting in HUS and kidney failure in some cases. In order to identify the specific *stx* genes that can increase virulence, *stx2* subtypes (*stx2f, stx2c, stx2e,* and *stx2d*) in the clinical and food strains were screened. The majority of strains carried 26% of the *stx2e* gene, which correlated with previously reported data [40]. The Stx2e subtype is generally not associated with serious human illness. Human infections linked to Stx2e producing *E. coli* generally cause asymptomatic infections or mild diarrhea [41]. Among the other subtypes, the *stx2c* gene was present in one clinical strain (2%), while the *stx2d* and *stx2f* genes were absent in all the strains tested. In contrast to our study, a previous study by Melton-Celsa et al. showed *stx2d* as a potent toxin which could lead to severe symptoms such as HC and HUS [10]. In addition, the STEC strains were also screened for the *hlyA* gene (α-hemolysin), which produces pores in the cytoplasmic membranes of eukaryotic cells, causing their death. It has been suggested that hlyA may increase the virulence of extraintestinal pathogenic *E. coli* [10,42]. In agreement with previous findings, data of the present study demonstrated that the *hlyA* gene was carried in 60% of the clinical strains and one food strain [43]. 

The presence of other toxin genes that may play roles in STEC pathogenesis was also evaluated in this study. *Irp2*, *nleB*, *eaf*, *eaf1*, *pagC*. and *sen* genes are located within high-pathogenicity islands (HPAIs) [44]. These genes affect the pathogenicity of STEC, and thus it is important to also screen for these genes. Moreover, mouse models have previously indicated that HPAIs increased *E. coli* virulence in extraintestinal infections [45]. In this study, these genes were present in most clinical isolates. Approximately 27% of the clinical isolates carried the *irp2* gene, while the food isolates did not. Additionally, 58% of the clinical strains and 34% of the food strains contained a conserved virulence gene profile. The *PagC* gene, located on an HPAI island, was found in the clinical isolates, which encodes an outer membrane protein, and it has also been reported in different Enterobacteriaceae that contribute to serum resistance [46]. In most studies, the *nleB* gene has been used to identify pathogens and differentiate between pathogenic strains and non-pathogenic strains [47]; 93% of the clinical strains were positive for the *nleB* gene and it was missing in most food strains, similar to the data reported in Argentina that compared the level of *nleB* in food, clinical, and animal strains [48].

The presence of *sen* in STEC strains indicates that horizontal transfer of virulence factors occurs on plasmids, HPAIs and other mobile genetic elements in bacteria belonging to different or similar species. A study in Brazil indicated that [49] 99% of these strains contained the *sen* gene.

The toxB protein encoded by the *toxB* gene is required for the full expression of adherence in the O157:H7 Sakai strain [50]. In the present study, almost 42% of the clinical strains showed presence of the *toxB* gene, which is similar to previous findings [12]. 

Only one strain from each of the isolated clinical and food strains was positive for *efa,* which is the most often adhesion protein described in enterohemorrhagic *E. coli* (EHEC) strains [51]. The *efa* gene is present in LEE locus and in all pathogens which produce A/E lesions. Data from a previous study showed 100% of food isolates and 75% of clinical strains carried the *efa1* gene [44]. The *efa-1* gene is very similar to *lifA*, a STEC gene encoding lymphostatin (LifA), which inhibits the reproduction of mitogen-activated lymphocytes and increases the synthesis of proinflammatory cytokines [52]. Previous studies have shown that *eae* was significantly associated with a higher risk for HUS development. The current findings show that 85% of the clinical strains and 66% of the food stains carry this gene, which is responsible for HUS [10]. Inc typing is an important method because there is a specific connection between Inc type and the presence of virulence genes and bacteriocins [53,54,55,56]. In this study, most of the strains carrying plasmid replicons have the highest number of virulence genes. Among the clinical strains, 73% and 32% were carrying the X gene and FIB, while W, HI1, I1 and B/O genes were found between 13% and 2.5% of the clinical strains. However, W, the most prevalent gene, was present in 83% of the food strains and 33% have B/O plasmid replicons. Plasmid found in the food strains were different from those in the clinical strains. 

In this study, the data demonstrated the prevalence of multidrug resistance among different strains and at least five virulence genes present in each strain; in addition, plasmid profiles and PFGE fingerprinting showed the genotypes of these strains. The combination of both PFGE fingerprinting and plasmid profiling can be a useful and reliable tool for discriminating STEC strains from different sources [25]. This analysis indicated that STEC strains from clinical and food sources showed dissimilarities including the genotypes and phenotypes, which play an important role during pathogenesis, and thus impact public health. In USA, from 2006 to 2019, at least two outbreaks caused by STEC O157 and, in 2020, one outbreak by other STEC serotype have been reported by CDC. It is important to understand the molecular epidemiology and prevalence of serotypes among clinical and food isolates. This study demonstrated that clinical isolates showed increased prevalence of virulence factors compared to food isolates and most of these factors were encoded in the modified mobile genetic elements. These findings will improve the understanding of pathogenic phenotypes and genotypes of these bacteria with outbreak potential. Further investigations are important to better understand the significance of different serotypes of STEC, their genotypes of pathogenicity, profiles of drug resistance, and the spread of virulence genes among the strains. These findings aim to reduce the entry of these bacteria into the U.S. food chain, and thus positively impact public health.

## Figures and Tables

**Table 1:** Primers used in the PCR for detection of virulence genes in STEC.

Gene	Sequence of Nucleotides	Target Gene	Size (bp)
*eae*	F: ACGTTGCAGCATGGGTAACTC R: GATCGGCAACAGTTTCACCTG	*eae*	(818)
*hlyA*	F: GGTGCAGCAGAAAAAGTTGTAG R: TCTCGCCTGATAGTGTTTGGTA	*hlyA*	(1551)
*stx1*	F: TCTCAGTGGGCGTTCTTATG R: TACCCCCTCAACTGCTAATA	*stx1*	(338)
*stx2*	F: GCGGTTTTATTTGCATTAGC R: TCCCGTCAACCTTCACTGTA	*stx2*	(115)
*stx2f*	F: TGTCTTCAGCATCTTATGCAG R: CATGATTAATTACTGAAACAGAAAC	*stx2f*	(150)
*stx2c*	F: GCGGTTTTATTTGCATTAGT R: AGTACTCTTTTCCGGCCACT	*stx2c*	(124)
*stx2e*	F: ATGAAGTGTATATTGTTAAAGTGGA R: AGCCACATATAAATTATTTCGT	*stx2e*	(303)
*stx2d*	F: GGTAAAATTGAGTTCTCTAAGTAT R: CAGCAAATCCTGAACCTGACG	*stx2d*	(175)
*nel B*	F: GGAAGTTTGTTTACAGAGACG R: AAAATGCCGCTTGATACC	*nleB*	(297)
*pagC*	F: ATGAGTGGTTCAAGACTGG R: CCAACTCCAACAGTAAATCC	*pagC*	(521)
*sen*	F: GGATGGAACCATACCTGG R: CGCAATCAATTGCTAATGC	*sen*	(551)
*efa*	F: CTCCCAGAGATAATTTTGAGG R: CAACTGTATGCGAATAGTACTC	*efa1*	(504)
*efa1*	F: CTGTCAGACGATGACATTGG R: GAAGGATGGGCATTGTGTC	*efa1*	(547)
*irp2*	R: GAAGGATGGGCATTGTGTC F: AAGGATTCGCTGTTACCGGAC	*irp2*	(280)
*toxB*	F: TGGCCTTGCGCTCTATAAGAACCT R: ACCACGCCGTGAGAATAATGTCCA	*toxB*	(823) c

**Table 2:** Primers used in the PCR for detection of plasmid replicon typing in STEC.

Replicon	Sequence of Nucleotides	Target Site	Size (bp)
*HI1*	F-GGAGCGATGGATTACTTCAGTAC R-TGCCGTTTCACCTCGTGAGTA	parA-parB	471
*HI2*	F-TTTCTCCTGAGTCACCTGTTAACAC R-GGCTCACTACCGTTGTCATCCT	iterons	644
*I1*	F-CGAAAGCCGGACGGCAGAA R-TCGTCGTTCCGCCAAGTTCGT	RNAI	139
*X*	F-AACCTTAGAGGCTATTTAAGTTGCTGAT R-TGAGAGTCAATTTTTATCTCATGTTTTAGC	Ori γ	376
*L/M*	F-GGATGAAAACTATCAGCATCTGAAG R-CTGCAGGGGCGATTCTTTAGG	rep A, B, C	785
*N*	F-GTCTAACGAGCTTACCGAAG R-GTTTCAACTCTGCCAAGTTC	rep A	559
*FIA*	F-CCATGCTGGTTCTAGAGAAGGTG R-GTATATCCTTACTGGCTTCCGCAG	iterons	462
*FIIB*	F-GGAGTTCTGACACACGATTTTCTG R-CTCCCGTCGCTTCAGGGCATT	rep A	702
*W*	F-CCTAAGAACAACAAAGCCCCCG R-GGTGCGCGGCATAGAACCGT	rep A	242
*FIC*	F-GTGAACTGGCAGATGAGGAAGG R-TTCTCCTCGTCGCCAAACTAGAT	rep A2	262
*A/C*	F-GAGAACCAAAGACAAAGACCTGGA R-ACGACAAACCTGAATTGCCTCCTT	rep A	465
*T*	F-TTGGCCTGTTTGTGCCTAAACCAT R-CGTTGATTACACTTAGCTTTGGAC	rep A	750
*FIIA*	F-CTGTCGTAAGCTGATGGC R-CTCTGCCACAAACTTCAGC	rep A	270
*K/B*	F-GCGGTCCGGAAAGCCAGAAAAC R-TCTTTCACGAGCCCGCCAAA	RNAI	160
*B/O*	F-GCGGTCCGGAAAGCCAGAAAAC R-TCTGCGTTCCGCCAAGTTCGA	RNAI	159
*Y*	F-AATTCAAACAACACTGTGCAGCCTG R-CGAGAATGGACGATTACAAAACTTT	repA	765
*P*	F-CTATGGCCCTGCAAACGCGCCAGAAA R-TCACGCGCCAGGGCGCAGCC	iterons	534
*Rep*	F-TGATCGTTTAAGGAATTTTG R-GAAGATCAGTCACACCATCC	RNAI/repA	270

## Data Availability

Not applicable.
